# Indicators for pulmonary hypertension in dogs with degenerative mitral valve disease

**DOI:** 10.14202/vetworld.2023.2515-2520

**Published:** 2023-12-25

**Authors:** Pasika Chalermpromma, Sirilak Disatian Surachetpong

**Affiliations:** Department of Veterinary Medicine, Faculty of Veterinary Science, Chulalongkorn University, Bangkok 10330, Thailand

**Keywords:** canine, degenerative mitral valve disease, pulmonary hypertension

## Abstract

**Background and Aim:**

Pulmonary hypertension (PH) is a common complication of degenerative mitral valve disease (DMVD) in dogs. This study aimed to determine PH indicators in dogs with stage C DMVD through thoracic radiography and M-mode and 2-dimensional echocardiography.

**Materials and Methods:**

A retrospective study of 133 dogs with DMVD stage C was conducted. The patients were divided into two groups: DMVD and DMVD with PH. Characteristics, thoracic radiographic findings, and echocardiographic variables were recorded for analysis.

**Results:**

Pulmonary hypertension was diagnosed in 56 dogs, with a prevalence of 42.1%. Univariable logistic regression analysis revealed associations among PH and right-sided heart enlargement, pulmonary artery enlargement, left ventricular size, and percent fractional shortening. Multivariable logistic regression analysis demonstrated that pulmonary artery enlargement (odds ratio [OR]: 5.96; 95% confidence interval [CI]: 1.45–24.54; p = 0.014) and decreased left ventricular size (OR: 0.02; 95%CI: 0.003–0.13; p = 0.001) were significantly associated with PH. The accuracy of predicting PH in dogs at DMVD stage C using pulmonary artery enlargement combined with decreased left ventricular size was moderate (area under the curve [AUC] 0.77; 95%CI: 0.68–0.86).

**Conclusion:**

Pulmonary hypertension is common in dogs with DMVD stage C. The presence of pulmonary artery enlargement assessed by radiography and decreased left ventricular size assessed by echocardiography, were associated with PH and may serve as predictors of PH in DMVD stage C.

## Introduction

Pulmonary hypertension (PH) is defined as an abnormal increase in pulmonary arterial pressure (PAP) that can occur in numerous diseases [1]. It is a common complication of degenerative mitral valve disease (DMVD), the most common acquired cardiac disease in dogs, accounting for approximately 75% of all heart diseases [2]. More than 90% of DMVD cases occur in small-breed dogs. According to the American College of Veterinary Internal Medicine (ACVIM) consensus guideline for the diagnosis and treatment of DMVD in dogs [3], DMVD is classified into four groups, comprising stages A, B, C, and D. The prevalence of PH due to DMVD in dogs ranges from 14% to 65% [4–7]. Pulmonary hypertension is commonly found in dogs with DMVD stages C and D [3]. Dogs with DMVD with PH have a poorer prognosis and a shorter survival time than those without PH [8].

Dogs with DMVD and PH may be asymptomatic or have clinical signs consistent with pulmonary edema and right-sided congestive heart failure at an advanced stage [4,9]. Measurement of the mean PAP is one diagnostic method for PH. Right heart catheterization (RHC) is the gold standard method for directly measuring PAP in humans [1]. However, RHC is rarely used in dogs due to its invasive nature. Spectral Doppler echocardiography is more commonly employed in veterinary medicine to estimate PAP by measuring the velocity of tricuspid regurgitation (TR) [4]. The ACVIM consensus statement guideline for the diagnosis of PH in dogs recommends echocardiography as an early diagnostic test to assess the probability of PH [7]. However, there are no specific echocardiographic guidelines for PH in dogs, and the echocardiographic examination, particularly spectral Doppler echocardiography, is considered to be at the best practice level of the echocardiographic protocol, which should be conducted by experienced cardiologists [10]. Therefore, general practitioners may not be fully aware of the development of PH in dogs with DMVD. While general practitioners may not be able to conduct Doppler studies for measuring estimated PAP, they can still observe initial changes in cardiac structure indicative of PH by evaluating M-mode and two-dimensional echocardiography. These structural changes could serve as the initial clues for diagnosis PH.

To the best of our knowledge, factors associated with PH in dogs with stage C DMVD have not yet been published. The present study aimed to determine PH indicators in dogs with stage C DMVD through thoracic radiography, M-mode, and two-dimensional echocardiography. The information from this study will assist general practitioners in the initial assessment of PH in dogs with stage C DMVD.

## Materials and Methods

### Ethical approval

The ethical approval is not necessary due to the retrospective design of the study.

### Study period and location

A retrospective study was conducted from August 2015 to July 2020 to collect medical records from the Small Animal Teaching Hospital, Faculty of Veterinary Science, Chulalongkorn University, Thailand.

### Animals

Inclusion criteria comprised small breed dogs diagnosed with stage C DMVD. The diagnosis of DMVD stage C was based on the 2019 ACVIM guidelines, which include a vertebral heart score (VHS) >10.5 determined by thoracic radiography. Echocardiographic findings included mitral valve thickening and mitral regurgitation (MR), left atrial to aorta dimension ratio (LA/Ao) in early diastole >1.6, and left ventricular internal diameter in diastole normalized by the Cornell allometric scale method (LVIDDN) >1.7. Signs of heart failure, assessed by thoracic radiography, had to be observed in dogs [3]. Dogs with abnormalities or diseases, such as infectious diseases, respiratory diseases, and other systemic conditions, were excluded from this study. Dogs diagnosed with DMVD stage C were divided into two groups: DMVD and DMVD with PH. Pulmonary hypertension was defined by a peak velocity of TR of more than 3 m/s or a TR pressure gradient of more than 36 mmHg as determined by Doppler echocardiography in the left parasternal apical 4-chamber view [7]. Characteristic variables, including breed, sex, age, weight, heart rate (HR), and blood pressure, were recorded.

### Thoracic radiography

Thoracic radiography was performed in two views: a lateral and a ventrodorsal (VD) view. The VHS, right heart enlargement, and pulmonary artery (PA) enlargement assessed by the thoracic radiography were recorded. Right heart enlargement was considered when there was increased sternal contact on the lateral view or an inverted D-shaped cardiac silhouette in the VD view. Main PA enlargement was identified as a focal bulge in the two o’clock position in the VD view. Peripheral PA enlargement was assessed subjectively by comparing the size of PA with an adjacent pulmonary vein or the width of the fourth rib in the lateral view or the ninth rib in the VD view [7,11].

### Echocardiography

An experienced investigator (SS) conducted echocardiography using an ultrasound machine (M9, Mindray, China) equipped with multifrequency 4–12 MHz phased-array transducers. All dogs were unsedated during the examination and were placed in right and left lateral recumbency positions. The characteristics of mitral and tricuspid valves were assessed using the right parasternal long-axis view. M-mode echocardiography was used to measure interventricular septum and left ventricular free wall thickness and left ventricle dimension in both systole and diastole. The M-mode echocardiographic values were normalized using Cornell’s allometric scaling method [12]: To assess left ventricular function, the percentage of fractional shortening (FS%) was calculated by measuring the percentage change in the left ventricular dimension [13]. The LA/Ao in early diastole was measured in the right parasternal short-axis view [3]. Color flow and spectral Doppler echocardiography were utilized to estimate aortic flow velocity, PA flow velocity, MR, and TR [13]. Right heart enlargement was subjectively assessed relative to the size of the left ventricle.

### Statistical analysis

Statistical analysis was performed using the statistical package for the social sciences version 28 (IBM Corp., NY, USA). Descriptive statistical analysis was conducted to determine the population characteristics. The Kolmogorov–Smirnov normality test was used to assess data distribution. Normally distributed continuous data are presented as mean ± standard deviation, while categorical variables are expressed as the number (%) of dogs. The independent-sample t-test was applied for continuous variables, and the Chi-square test or Fisher’s exact test was employed for categorical variables to compare DMVD dogs with and without PH. p < 0.05 was considered statistically significant.

Univariate logistic regression analysis was used to estimate the OR with 95% confidence interval (CI) to assess the association between PH and characteristic thoracic radiographic and echocardiographic variables. For model selection, variables with p < 0.1 from the univariable logistic regression analysis were included in the full multivariable logistic regression model. The backward elimination method was used for multivariable logistic regression analysis. The Hosmer–Lemeshow goodness-of-fit test was used to assess the goodness-of-fit for the final multivariable logistic regression model [14]. p < 0.05 was considered indicative of statistical significance.

Subsequently, the model for the risk of developing PH obtained from the multivariable logistic regression analysis was further analyzed for predictive ability using receiver operating characteristic (ROC) curve and area under the curve (AUC) analysis. A model with an AUC value > 0.8 or between 0.7 and 0.8 was considered to have good and moderate predictive ability, respectively [14].

## Results

Data from 133 dogs with stage C DMVD were included in the study. The DMVD and DMVD with PH groups comprised 77 and 56 dogs, respectively. In this study, the prevalence of PH secondary to DMVD stage C was 42.1%. Table-1 summarizes the descriptive data of the dogs recruited for the study. Chi-square analysis indicated that right heart and PA enlargement on thoracic radiography (p = 0.02 and 0.002, respectively) and echocardiography (p < 0.001) were associated with PH in dogs with stage C DMVD. Comparisons of continuous variables between dogs with DMVD and those with PH showed decreased left ventricular diastolic diameter normalized to body weight (LVDDn) (p < 0.001) and left ventricular systolic diameter normalized to body weight (LVSDn) (p < 0.001) and increased FS% (p = 0.007) in dogs with DMVD and PH.

**Table-1:** Descriptive data of 133 DMVD stage C dogs with and without PH.

Variables	Category	DMVD (n=77) (%)	DMVD with PH (n=56)	p-value
Breed	Crossbred	13 (16.9)	7 (12.5)	0.485
	Purebred	64 (83.1)	49 (87.5)	
Sex	Male	49 (63.6)	30 (53.6)	0.243
	Female	28 (36.4)	26 (46.4)	
Age (years)		11.75 ± 2.88	12.20 ± 2.55	0.36
Weight (kg)		5.38 ± 2.06	5.73 ± 3.05	0.431
HR (bpm)		137.5 ± 27.97	145.95 ± 33.57	0.301
BP (mmHg)		131.70 ± 20.96	137.95 ± 30.12	0.328
Radiography				
VHS		11.74 ± 0.7	11.99 ± 0.89	0.088
RT enlargement	Yes	2 (2.9)	8 (15.4)	**0.02**
	No	66 (97.1)	44 (84.6)	
PA enlargement	Yes	3 (4.4)	12 (23.1)	**0.002**
	No	65 (95.6)	40 (76.9)	
Echocardiography				
RT enlargement	Yes	0 (0)	19 (33.9)	**<0.001**
	No	77 (100)	37 (66.1)	
LVDDn		2.01 ± 0.22	1.75 ± 0.4	**<0.001**
LVSDn		1.1 ± 0.23	0.85 ± 0.3	**<0.001**
IVSDn		0.45 ± 0.1	0.44 ± 0.8	0.321
IVSSn		0.64 ± 0.11	0.61 ± 0.12	0.267
LVWDn		0.39 ± 0.08	0.39 ± 0.11	0.806
LVWSn		0.7 ± 0.1	0.68 ± 0.13	0.476
LA (cm)		2.55 ± 0.5	2.54 ± 0.66	0.932
Ao (cm)		1.22 ± 0.17	1.17 ± 0.23	0.337
LA/Ao		2.15 ± 0.43	2.17 ± 0.4	0.713
FS (%)		45.62 ± 7.42	49.48 ± 8.86	**0.007**
PA flow velocity (m/s)		0.70 ± 0.23	0.85 ± 0.54	0.136
Ao flow velocity (m/s)		0.98 ± 0.29	1.10 ± 0.29	0.103
MR (m/s)		5.14 ± 0.64	4.97 ± 0.59	0.31
MV E peak (ms)		1.26 ± 0.31	1.40 ± 0.79	0.212
MV A peak (ms)		0.93 ± 0.3	0.85 ± 0.21	0.288
MV E/A		1.54 ± 0.40	1.70 ± 0.91	0.441
TR (m/s)		2.51 ± 0.39	3.83 ± 0.71	**<0.001**
TR PG (mmHg)		25.74 ± 7.6	60.57 ± 24.46	**<0.001**

The bold values indicate statistical significance, HR=Heart rate, BP=Blood pressure, VHS=Vertebral heart score, RT enlargement=Right-sided heart enlargement, PA enlargement=Pulmonary artery enlargement, LVDDn=Left ventricular diastolic diameter normalized to body weight, LVSDn=Left ventricular systolic diameter normalized to body weight, IVSDn=Interventricular septum thickness in diastole normalized to body weight, IVSSn=Interventricular septum thickness in systole normalized to body weight, LVWDn=Left ventricular free wall thickness in diastole normalized to body weight, LVWSn=Left ventricular free wall thickness in systole normalized to body weight, LA=Left atrial diameter, Ao=Aorta diameter, LA/Ao=Left atrial to aortic ratio, FS=Fractional shortening, PA flow velocity=Pulmonary artery flow velocity, Ao flow velocity=Aortic flow velocity MR=Mitral regurgitation flow velocity, MV E peak=Mitral valve peak velocity of early diastolic transmitral flow wave (E wave), MV A peak=Mitral valve peak velocity of early diastolic transmitral flow wave (A wave), MV E/A=Mitral valve peak velocity ratio between peak E and peak A, TR=Tricuspid regurgitation flow velocity, TR PG=Tricuspid regurgitation pressure gradient, DMVD=Degenerative mitral valve disease, PH=Pulmonary hypertension

Univariable logistic regression analysis demonstrated that PH was associated with the presence of right heart and PA enlargement on thoracic radiography, LVDDn, LVSDn, and the percentage of fractional shortening (%FS) (all p < 0.05) (Table-2).

**Table-2:** The univariable logistic regression analysis of factors associated to PH in DMVD stage C dogs.

Variables	Odds ratio (95% CI)	p-value
Age (years)	1.06 (0.94–1.21)	0.358
Weight (kg)	1.06 (0.92–1.21)	0.43
HR (BPM)	1.01 (0.99–1.03)	0.298
BP (mmHg)	1.01 (0.99–1.03)	0.352
Radiography		
VHS	1.52 (0.94–2.47)	0.091
RT enlargement		
Yes	6.00 (1.22–29.59)	**0.028**
No	1.00	
PA enlargement		
Yes	6.50 (1.73–24.46)	**0.006**
No	1.00	
Echocardiography		
LVDDn	0.04 (0.01–0.21)	**<0.001**
LVSDn	0.02 (0.003–0.12)	**<0.001**
IVSDn	0.14 (0.003–6.87)	0.32
IVSSn	0.1 (0.002–5.78)	0.26
LVWDn	0.63 (0.01–25.18)	0.805
LVWSn	0.28 (0.01–13.07)	0.514
LA (cm)	0.97 (0.45–2.1)	0.931
Ao (cm)	0.34 (0.04–3.06)	0.334
LA/Ao	1.17 (0.51–2.69)	0.711
FS (%)	1.06 (1.02–1.11)	**0.009**
PA flow velocity (m/s)	6.19 (0.51–75.73)	0.154
Ao flow velocity (m/s)	5.5 (0.68–44.69)	0.111
MR (m/s)	0.64 (0.27–1.51)	0.306
MV E peak (ms)	1.71 (0.68–4.32)	0.257
MV A peak (ms)	0.28 (0.03–2.4)	0.244
MV E/A	1.4 (0.59–3.33)	0.477

The bold values indicate statistical significance, HR=Heart rate, BP=Blood pressure, VHS=Vertebral heart score, RT enlargement=Right-sided heart enlargement, PA enlargement=Pulmonary artery enlargement, LVDDn=Left ventricular diastolic diameter normalized to body weight, LVSDn=Left ventricular systolic diameter normalized to body weight, IVSDn=Interventricular septum thickness in diastole normalized to body weight, IVSSn=Interventricular septum thickness in systole normalized to body weight, LVWDn=Left ventricular free wall thickness in diastole normalized to body weight, LVWSn=Left ventricular free wall thickness in systole normalized to body weight, LA=Left atrial diameter, Ao=Aorta diameter, LA/Ao=Left atrial to aortic ratio, FS=Fractional shortening, PA flow velocity=Pulmonary artery flow velocity, Ao flow velocity=Aortic flow velocity MR=Mitral regurgitation flow velocity, MV E peak=Mitral valve peak velocity of early diastolic transmitral flow wave (E wave), MV A peak=Mitral valve peak velocity of early diastolic transmitral flow wave (A wave), MV E/A=Mitral valve peak velocity ratio between peak E and peak A, CI=Confidence interval, DMVD=Degenerative mitral valve disease, PH=Pulmonary hypertension

Multivariable logistic regression analysis showed that the presence of PA enlargement (p = 0.014) and LVSDn (p < 0.001) was significantly associated with the risk of developing PH (summarized in Table-3).

**Table-3:** The multivariable logistic regression analysis of factors associated to PH in DMVD stage C dogs.

Variables	Odds ratio (95% CI)	p-value
Radiography		
PA enlargement		
Yes	5.96 (1.45–24.54)	0.014
No	1.00	
Echocardiography		
LVSDn	0.02 (0.003–0.13)	<0.001

The bold values indicate statistical significance, PA=Pulmonary artery, LVSDn=Left ventricular systolic diameter normalized to body weight, CI=Confidence interval, DMVD=Degenerative mitral valve disease, PH=Pulmonary hypertension

The model derived from the multivariable logistic regression analysis was further analyzed using the ROC curve, and the AUC was 0.77 (95% CI 0.68–0.86).

## Discussion

This study demonstrated a frequent association between PH and DMVD in dogs. The presence of right heart and PA enlargement on thoracic radiography and a decrease in left ventricular size assessed by echocardiography were associated with the development of PH in dogs with stage C DMVD.

Although PH can result from several diseases, it is a common finding associated with the left heart disease both in humans and dogs. The prevalence of PH in patients with the left-sided heart disease is approximately 60%–80% in humans. In the previous studies, the prevalence of PH in dogs with mitral valve disease ranged from 14% to 65% [4–7]. PH was more common in dogs with stage C DMVD compared with B2 DMVD [8]. The prevalence of PH in dogs with stage C DMVD was approximately 42.1%, suggesting that PH is common in dogs with left-sided congestive heart failure, particularly those with DMVD.

The results of this study demonstrated that thoracic radiography could provide important information about the development of PH in dogs with DMVD. According to the ACVIM consensus statement guideline for the diagnosis of PH in dogs, further echocardiographic examination is recommended to assess PH in dogs with radiographic findings of dilated or tortuous PA and right heart enlargement with an inverted D shape [7]. In addition, radiography can identify signs of right-sided heart failure, such as pleural effusion, caudal vena cava enlargement, hepatomegaly, and ascites [15]. In a previous study, common radiographic findings in dogs with PH included cardiomegaly, right ventricular enlargement, cranial and caudal lobar PA enlargement, and pleural effusions [16]. Univariable logistic regression analysis in the present study showed that right heart and PA enlargement were associated with PH in dogs with stage C DMVD. However, multivariable logistic regression showed that only PA enlargement was associated with PH in dogs with stage C DMVD. This finding suggests that radiographic PA enlargement may predict PH in dogs with stage C DMVD. Therefore, further investigation is needed to assess the development of PH whenever dogs with DMVD, who frequently exhibit pulmonary vein enlargement secondary to pulmonary vein congestion and increased left atrial pressure, show signs of PA enlargement.

This study found that right heart enlargement, assessed subjectively by radiography and echocardiography, was associated with PH. A previous study also demonstrated an increase in right heart size and the presence of right heart failure which were associated with an increased risk of death in dogs with PH [17]. Another study suggested that signs of increased right ventricular and PA pressure, including flattening of the interventricular septum and right ventricular dilation or hypertrophy, were observed in dogs with moderate to severe PH [18]. Similarly, a previous study found an increase in the right ventricular end-diastolic internal diameter (RVIDDn) corrected for body weight in DMVD dogs with PH with a TR pressure gradient 55 mmHg or higher [19]. In our study, 19 dogs with DMVD and PH had right heart enlargement on echocardiography. Among them, most dogs (13 out of 19 dogs) had a TR pressure gradient >55 mmHg. These findings suggest that the presence of right heart enlargement increases with the severity of the TR pressure gradient. Therefore, subjective assessment of right-sided heart may be limited in detecting severe PH only.

In this study, left ventricular size decreased in dogs with DMVD and PH. In dogs with stage C DMVD, cardiac remodeling is already present, and echocardiographic findings show enlargement of the left atrium (LA/Ao > 1.6) or left ventricle (LVDDN > 1.7) [3]. All dogs in this study had LA/Ao values > 1.6 and radiographic findings were consistent with heart failure. This study showed a significant decrease in the left ventricular diameter in dogs with DMVD and PH. The reduction in the left ventricular size may be attributed to impaired filling in dogs with PH [18] and chronic progressive left heart disease [7,20]. In a dog model of chronic embolic precapillary PH achieved through repeated microsphere injections into five laboratory beagles until their systolic PAP reached 50 mmHg, the echocardiographic indices for all dogs indicated a significant increase in normalized RVIDDn and a significant decrease and normalized left ventricular end-diastolic internal diameter compared to baseline values [21]. %FS was significantly increased in dogs with DMVD and PH in this study. In general, left ventricular %FS is used to indicate systolic function, with the normal value of %FS in dogs ranging from 33% to 46% [22]. %FS can be calculated as [(LVDD-LVSD)/LVDD] × 100 [23]. The mean value of %FS in PH dogs with stage C DMVD in this study was 49.48% ± 8.86%, which may be due to decreased left ventricular size rather than a true increase in the left ventricular systolic function. A previous report on the prognostic value of pulmonary vascular resistance in dogs with DMVD and detectable TR found no significant associations between the severity of PH and the FS% [24].

Based on the results of multivariable logistic regression, PA enlargement assessed by radiography and decreased left ventricular size determined by echocardiography was associated with PH in dogs with stage C DMVD. The model derived from the multivariable logistic regression analysis was further analyzed by the ROC curve, showing an AUC of 0.77, interpreted as moderate performance. Therefore, subjective assessment of PA size on radiographs and decreased left ventricular size on echocardiography may offer valuable clues for diagnosing PH in dogs with stage C DMVD, particularly for primary care veterinarians.

This study had some limitations due to its retrospective design, which may have introduced some missing data affecting the statistical analysis. In addition, the use of echocardiography as a non-invasive diagnostic tool to assess PH based on TR is another limitation. The number of dogs with PH included in this study may not represent the overall population. Furthermore, the subjective assessment of right ventricular enlargement may vary depending on experience of investigators.

## Conclusion

PH is common in dogs with stage C DMVD. The presence of pulmonary enlargement and a decreased left ventricular size were associated with PH in dogs with stage C DMVD. The identification of these features may serve to remind and enhance veterinarians’ awareness of the potential development of PH in dogs with DMVD. Further, investigation by cardiologists should be conducted to confirm and evaluate the severity of PH for improved management and prognosis.

## Authors’ Contributions

SDS: Designed and supervised the study and drafted and revised the manuscript. PC: Collected and analyzed data and drafted and revised the manuscript. Boh authors have read, reviewed, and approved the final manuscript.
